# Evaluation of cell disruption methods for protein and coenzyme Q10 quantification in purple non-sulfur bacteria

**DOI:** 10.3389/fmicb.2024.1324099

**Published:** 2024-03-07

**Authors:** Ojima Z. Wada, Naim Rashid, Patrick Wijten, Paul Thornalley, Gordon Mckay, Hamish R. Mackey

**Affiliations:** ^1^Division of Sustainable Development, College of Science and Engineering, Hamad Bin Khalifa University, Qatar Foundation, Doha, Qatar; ^2^Department of Water Resources Engineering & Management, National University of Science and Technology (NUST), Islamabad, Pakistan; ^3^Qatar Biomedical Research Institute, Qatar Foundation, Doha, Qatar; ^4^Department of Civil and Natural Resources Engineering, University of Canterbury, Christchurch, New Zealand

**Keywords:** single cell protein, anoxygenic phototrophic bacteria, protein extraction, microbial protein, resource recovery

## Abstract

A recent focus has been on the recovery of single-cell protein and other nutritionally valuable bioproducts, such as Coenzyme Q10 (CoQ10) from purple non-sulfur bacteria (PNSB) biomass following wastewater treatment. However, due to PNSB’s peculiar cell envelope (e.g., increased membrane cross-section for energy transduction) and relatively smaller cell size compared to well-studied microbial protein sources like yeast and microalgae, the effectiveness of common cell disruption methods for protein quantification from PNSB may differ. Thus, this study examines the efficiency of selected chemical (NaOH and EDTA), mechanical (homogenization and bead milling), physical (thermal and bath/probe sonication), and combined chemical–mechanical/physical treatment techniques on the PNSB cell lysis. PNSB biomass was recovered from the treatment of gas-to-liquid process water. Biomass protein and CoQ10 contents were quantified based on extraction efficiency. Considering single-treatment techniques, bead milling resulted in the best protein yields (*p* < 0.001), with the other techniques resulting in poor yields. However, the NaOH-assisted sonication (combined chemical/physical treatment technique) resulted in similar protein recovery (*p* = 1.00) with bead milling, with the former having a better amino acid profile. For example, close to 50% of the amino acids, such as sensitive ones like tryptophan, threonine, cystine, and methionine, were detected in higher concentrations in NaOH-assisted sonication (>10% relative difference) compared to bead-milling due to its less disruptive nature and improved solubility of amino acids in alkaline conditions. Overall, PNSB required more intensive protein extraction techniques than were reported to be effective on other single-cell organisms. NaOH was the preferred chemical for chemical-aided mechanical/physical extraction as EDTA was observed to interfere with the Lowry protein kit, resulting in significantly lower concentrations. However, EDTA was the preferred chemical agent for CoQ10 extraction and quantification. CoQ10 extraction efficiency was also suspected to be adversely influenced by pH and temperature.

## 1 Introduction

In the past decade, there has been a growing interest to reduce the pressure on scarce natural resources like fresh water, arable land and forage fish by exploiting microbial biotechnologies to substitute conventional protein sources like soybeans (Santillan et al., 2024). Early single-cell protein (SCP) biotechnologies have included yeasts and microalgae (Wada et al., 2022). More recently, SCP derived from purple non-sulfur bacteria (PNSB) has emerged as a more desirable protein source due to its comparatively higher biomass protein content, carotenoid and coenzyme Q10 (CoQ10) content, high nutrient recovery rate, metabolic versatility and anti-pathogen properties (George et al., 2020; Koukoumaki et al., 2024).

Methods for disrupting cells to accurately extract and quantify biomass protein content have been extensively explored for microalgae and yeast biotechnologies (Halim et al., 2022; Rahman et al., 2022; Gautério et al., 2023). These cellular disruption techniques can be broadly classified into mechanical treatment (such as bead milling and homogenization), physical treatment (heating and sonication) and chemical/biological treatment (alkali, acids, and enzymes) (Soto-Sierra et al., 2018). Mechanical treatment breaches the cell envelope using shear force and is regarded as a non-selective lysis method (Liu et al., 2016). It is the most common method due to its comparatively higher lysing efficiency (Islam et al., 2017). Non-mechanical treatment techniques are gentler and more targeted, thereby increasing the selectivity of cellular constituents (Liu et al., 2016).

The effectiveness of these cell lysis techniques is dependent on the composition of the layers enclosing the cell and the size of the microbes of interest (Burden, 2012; Li et al., 2020). For example, Mielko et al. demonstrated that the optimal extraction method varied between six different bacteria belonging to both gram positive-and gram-negative cell wall groups (Mielko et al., 2021). Similar reports exist for microalgae, where some species have simple cell membranes consisting of mostly phospholipid bilayer and proteins, while others have additional layers of intracellular or/and extracellular materials leading to fundamental differences in preferred cell disruption methods (D’Hondt et al., 2017).

Compared to yeast, bacteria have a fundamentally different cell wall composition. Yeast’s cell wall consists of chitin, layers of mannoproteins and fibrous glucans, making them more difficult to breach than bacteria’s peptidoglycan layers (Utama et al., 2023). PNSB are gram-negative bacteria. Their cell envelope consists of the cytoplasmic membrane holding the cell contents; the peptidoglycan layer, which consists of lipoproteins, phospholipids, porin proteins, lipopolysaccharide, and other proteins; and an external outer membrane that provides mechanical strength (Weckesser et al., 1995; Rojas et al., 2018). This enables gram-negative bacteria to withstand lysozyme cell lysis better than gram-positive bacteria (Naveed et al., 2023). Moreover, photosynthetic bacteria like PNSB have an additional intracytoplasmic membrane (ICM) adjoined to the inner membrane to facilitate energy harvest from light, potentially increasing the cells’ resistance to cellular disruption (Oikonomou and Jensen, 2021). Even among phototrophs, there are differences in the cell envelope; for example, algae and cyanobacteria’s membrane lipid layer consists mainly of glycolipids, which are reportedly vulnerable to heat stress, while PNSB’s membrane contains mostly phospholipids like phosphatidylglycerol and phosphatidylethanolamine which are reportedly heat tolerant (Su et al., 2009; Nagatsuma et al., 2019). In addition, PNSB has a smaller cell size (0.3–1.7 μm) compared to commonly explored SCP microbes like *Saccharomyces cerevisiae* (8–10 μm), *Spirulina* sp. (>100 μm) and *Chlorella vulgaris* (2–10 μm) (Becker, 2013; Novak et al., 2017; Vander Wiel et al., 2017; Zakhartsev and Reuss, 2018; Teiba et al., 2020).

Despite the extensive research on cell disruption techniques for microalgae and yeast, there remains a significant gap in understanding the effectiveness of these methods when applied to PNSB, given their unique cellular architecture and size (Madigan and Jung, 2009). The gram-negative membrane composition, additional intracytoplasmic membrane, and smaller cell size provide possible resistance to lysozomes, heat and mechanical stress. Therefore, this study aims to fill this crucial research gap by evaluating the effectiveness of various cell disruption methods, including mechanical (homogenization with mortar and pestle, bead milling), physical (thermal and sonication), chemical (alkali and EDTA), and combined treatments, specifically for quantifying protein and CoQ10 content in PNSB biomass. As PNSB biotechnology is evolving to become a more commonly explored choice for wastewater treatment and bioresource recovery of microbial protein (Wada O. Z. et al., 2023), optimizing characterization methods specifically for this organism type is crucial.

## 2 Materials and methods

### 2.1 Biomass production and processing

Using 1 L Duran bottles, a PNSB-dominated mixed culture (50 mg/L) was inoculated in organic-rich industrial process water originating from the Fischer-Tropsch process with a chemical oxygen demand (COD) of 6 g/L. The process water was augmented with nutrients (NH_4_Cl, KH_2_PO_4_), trace minerals and vitamin supplements. A mixed culture was used since there is a growing trend in the integration of SCP production with wastewater treatment, where PNSB-enriched systems are of interest due to the ability to enrich the target organism under non-axenic conditions easily (Delamare-Deboutteville et al., 2019; Wada O. et al., 2023). PNSB dominance was confirmed via the distinctive reddish color of the culture and microbial community analysis using next-generation sequencing. *Rhodopseudomonas* sp. had an OTU abundance of 35%, accounting for over one-third of the culture, while the remainder mostly consisted of heterotrophic anaerobic bacteria like *Plaudibacter* sp. and *Lentimicrobium* sp. Continuous light was supplied using LED grow lamps transmitting ~75% of the spectral power within the 400–800 nm range at an irradiance of 18 W/m^2^. Mixing at 200 rpm was provided via magnetic stirring. At the end of the culture period, the cells had a biomass concentration of about 1.8 g/L and were harvested via centrifugation at 10,000 g (Sorvall Lynx 6000, ThermoScientific). The biomass was stored at-80°C until freeze-drying was performed (FreeZone 6, Labconco). The freeze-dried biomass was then homogenized using a mortar and pestle and aliquoted based on the cellular disruption methods examined.

### 2.2 Cellular disruption techniques

Eight different cellular disruption techniques successfully utilized for microalgae and *Escherichia coli* biomass processing, were examined. These can be categorized into mechanical, chemical, and combined mechanical/physical–chemical treatments. The mechanical treatment methods examined were homogenization and bead milling, and the physical treatment methods used were thermal lysis, bath sonication, and probe sonication. The chemical treatments were performed using either 0.04 M EDTA or 0.4 M NaOH. The combined chemical and physical/mechanical treatments trialed each combination of the incorporated methods (i.e., for each mechanical cell disruption method both a 0.04 M EDTA and 0.4 M NaOH chemical addition were tested separately). For mechanical and physical techniques, samples were suspended in distilled water before processing.

EDTA is commonly used to weaken the cell wall by increasing its permeability. In *E. coli*, optimal cellular lysis was achieved at 0.04 M EDTA (Anand et al., 2007); hence, this concentration was adopted. However, EDTA has been identified as an interfering substance for the modified Lowry protein assay in concentrations above 1 mM (Thermo Scientific, 2020). NaOH has also been identified as an effective lysis agent and has been successfully used for microalgae studies (Safi et al., 2014). Microalgae studies have recommended NaOH molarity >0.25 M and <1.0 M to avoid incomplete extraction and protein loss (Rausch, 1981; Safi et al., 2014). Besides its role in membrane permeation, high alkalinity (>pH 12) has been reported to fully solubilize biomass protein (Gerde et al., 2013). A summary of all treatment techniques examined is provided in Table 1.

**Table 1:** Summary of cellular disruption techniques applied.

Techniques	Key process/method	Treatment solutions	Treatment variations
**Mechanical**			
Crude homogenization	Mortar and pestle	Distilled water, EDTA and NaOH	Instantaneous mixing and 1 h mixing
Bead beating	Bead milling at 2,000 rpm	Distilled water, EDTA and NaOH	
**Physical**			
Thermal	Block digester at 90°C	Distilled water, EDTA and NaOH	Instantaneous mixing and 1 h mixing
Probe sonication	Frequency of 20 kHz and 13 mm tip diameter	Distilled water, EDTA and NaOH	0.5 h sonication and 1 h sonication
Bath sonication	Frequency of 40 kHz and 80°C temperature	Distilled water, EDTA and NaOH	0.5 h sonication and 1 h sonication
**Chemical**			
0.04 M EDTA	Mixing for 2 h at 100 rpm	EDTA	
0.4 M NaOH	Mixing for 2 h at 100 rpm	NaOH	

For all samples, biomass was initially homogenized with a mortar and pestle before further processing with different cellular disruption methods. For all treatment groups, a biomass weight of 15 mg and solution volume of 10 mL was used to maintain extracted protein concentrations within the calibration curve limits (1,500 μg/mL) of the Lowry method. The effect of each treatment method was assessed in replicate. Details of each treatment process are provided below.

#### 2.2.1 Chemical treatment

This was performed by suspending 15 mg of homogenized biomass in 10 mL of 0.04 M EDTA (at pH 8) or 0.4 M NaOH. After suspension, the samples were vortexed and mixed at room temperature for 2 h at 100 rpm using a compact digital rocker (JIKT20022, ThermoScientific). This was to allow enough time for the chemicals to weaken the cell walls and induce lysis. The cells were subsequently centrifuged at 8,000 g, and the supernatant was collected for protein quantification.

#### 2.2.2 Thermal and chemical-assisted thermal treatment

A known weight of homogenized PNSB biomass (15 mg) was placed in glass vials. The glass vials were then separated into four different groups depending on the suspension solution employed. The cells were suspended in 10 mL of distilled/deionized water, 0.04 M EDTA or 0.4 M NaOH. The samples were thoroughly vortexed to ensure homogeneity. Using a compact digital rocker, all chemical treatment methods were also assessed for reaction (mixing) time, considering rapid (vortexing for 1 min) and extended (1 h at 100 rpm) mixing intensities. Thermal treatment was performed with a block digester (DRB 200, Hach). The samples were heated at 90°C for 1 h and left to cool at room temperature. After cooling, the samples were centrifuged at 8,000 g, and the supernatant was collected for protein quantification.

#### 2.2.3 Sonication and chemical-assisted sonication treatment

This method was examined by suspending 15 mg of biomass in 10 mL of distilled water, 0.04 M EDTA, or 0.4 M NaOH. After suspension, the samples were vortexed and mixed for 1 h, as described in Section 2.2.2. The efficiency of bath and probe sonication were compared. Bath sonication was performed at a frequency of 40 kHz and temperature of 80°C (WUC-D10H, Daihan Scientific). In comparison, probe sonication was performed at an output frequency of 20 kHz, and the probe had a tip diameter of 13 mm (Model 150VT Ultrasonic Homogenizer, Biologics Inc.). For all samples, sonication was performed for a duration of 1 h. The impact of shorter sonication duration (30 min) was also examined. After sonication the samples were left to cool at room temperature and centrifuged at 8,000 g before processing the supernatant for protein quantification.

#### 2.2.4 Bead milling and chemical-assisted bead milling

Biomass samples of 15 mg were suspended in 10 mL of distilled water, 0.04 M EDTA, or 0.4 M NaOH. The samples were vortexed for homogeneity and mixed for 1 h, as described earlier. Subsequently, the samples were transferred to a high-energy ball milling chamber (Emax, Retsch), which was filled to 65% capacity with 2 mm diameter stainless steel beads weighing 98.5 g (product 22.455.0010, Retsch). Bead milling was performed for 30 min at 2000 rpm. The maximum milling chamber temperature was set to 70°C. The bead miller automatically stopped to cool once the maximum temperature was attained. After milling, the samples were collected into a container, centrifuged at 8,000 g and processed for protein analysis.

### 2.3 Protein and amino acid quantification

The modified Lowry protein assay is one of the most referenced protein quantification methods. The assay is based on the ability of proteins to react with copper(II) sulfate and tartrate to yield copper-protein complexes. Upon reaction of the Cu-protein complex with Folin–Ciocalteu’s reagent, a reduction reaction occurs. The extent of reduction is directly proportional to the amount of chelated Cu complexes giving a blue color, which can be measured spectrophotometrically around 750 nm.

For this study, protein quantification was performed following the recommended protocol of the Pierce™ Modified Lowry protein kit 23240 (Thermo Scientific). In short, 10 protein standards were prepared at different concentrations using the reference albumin stock provided and distilled water as diluent. The Folin-Coicalteu reagent was prepared by diluting it in an equal portion of distilled water. Subsequently, 1 mL of Modified Lowry reagent was added to 0.2 mL of the standards and samples. After mixing and incubating, 100 μL of Folin-Coicalteu reagent was added to the standards and samples. The mixture was then vortexed, incubated for 30 min and analyzed using a UV-3600 plus UV–Vis–NIR spectrophotometer (Shimadzu) at 750 nm. After obtaining the readings, a standard curve was generated, and sample protein content was determined from the standard curve.

Subsequently, a 100 μg fraction of protein was subjected to thorough enzymatic hydrolysis in a controlled environment devoid of oxygen, utilizing a PAL sample autoprocessor (CTC Analytics). The resulting hydrolyzed protein was utilized for amino acid identification using LC–MS/MS, employing an Acquity UPLC system (Waters) coupled with a Quattro Premier tandem mass spectrometer, following the protocol described by Rabbani and Thornalley (2020).

### 2.4 CoQ10 quantification

CoQ10 detection was performed using the Human coenzyme Q10 ELISA kit MBS165643 (MyBiosource). The assay is based on the competitive inhibition enzyme immunoassay technique. In brief, five different standard concentrations were prepared by diluting with the provided sample diluent. Then, 50 μL of standards were added to the microwell plates, followed by 40 μL of samples. 10 μL of anti-CoQ10 antibody were then added to sample wells, after which 50 μL of streptavidin-horseradish peroxidase-conjugate to all wells. The microplate was covered and incubated at 37°C for 60 min before washing the wells using the provided wash buffer and aspirating five times. After aspirating, 50 μL of solution A and B were simultaneously added to the wells and incubated in the dark at 37°C for 10 min, followed by adding 50 μL of “stop solution.” Optical density measurements at 450 nm were taken using a Spark multimode microplate reader (Tecan). The standard curve was derived from the readings, and the sample concentrations were determined.

### 2.5 Statistical analysis

Descriptive analysis was performed using Microsoft Excel, while inferential statistics were performed using JASP 0.14.1.0. All inferential statistics were measured at the 95% confidence interval, with statistical significance at *p* < 0.05. After verifying the test assumptions’ validity, the independent *t*-test was used to determine if statistically significant differences existed between two groups and the one-way analysis of variance test when more than two groups were assessed. Due to the limited replicate number, a conservative Bonferroni *post-hoc* test was utilized. When Levene’s test was statistically significant, Welch’s test was used in place of ANOVA and the Games-Howell *post hoc* test was used in place of the Bonferroni *post hoc* test.

## 3 Results and discussion

### 3.1 Chemical treatment

Chemical treatments with alkali are often appraised due to their low energy use, ease of scaleup and cost efficiency (Hu and Bassi, 2020). The protein extraction performance of both chemical methods (EDTA and NaOH) on the PNSB biomass was significantly greater than the control treatment with distilled water (*p* < 0.001) but significantly less (*p* < 0.003) than all the combined chemical–mechanical treatments examined. The inefficiency of water has also been reported in microalgae studies (Gerde et al., 2013). The reduced extraction efficiency of chemical treatment compared to chemical-assisted mechanical treatments is probably because the chemicals cannot fully lyse the cells and release intracellular proteins. Their common mechanism of action is to make the cell wall more permeable for selective proteins to seep through, which can be enhanced through cellular disruption processes (Ren et al., 2007; Callejo-López et al., 2020). Incomplete extraction of total biomass protein has also been reported in other microalgae studies that utilized alkaline treatment in isolation (Rausch, 1981; Callejo-López et al., 2019, 2020). Additional physical/enzymatic treatment was employed to increase protein extraction efficiency. The EDTA treatment group (27.1 ± 0.1% biomass protein) had a slightly higher extraction efficiency compared to the NaOH group (23.0 ± 1.1% biomass protein). Similar results were obtained in studies that examined chemical methods for extracting protein and extracellular polymeric substances from *Arabidopsis* cells, *Rhodopseudomonas acidophila*, and activated sludge (Sheng et al., 2005; Liang et al., 2010; Tsugama et al., 2011). Other studies with *S. cerevisiae* have reported that solely using NaOH as a lysis agent only breaches the cell wall partially, therefore requiring additional treatment steps to ensure full cellular lysis (Vitaly, 2000; von der Haar, 2007). This implies that NaOH is probably more suitable when used with a mechanical cell disruption method, as affirmed in the subsequent sections. However, the downside of alkaline treatment is lysinoalanine formation, which results in the loss of the essential amino acid lysine and toxicity in consumption-related application (van den Berg et al., 2022; McKerchar et al., 2023).

### 3.2 Thermal treatment

The effect of thermal treatment alone and chemical-assisted thermal treatment was assessed under two conditions: rapid mixing (1 min vortexing) and extended mixing (1 h at 100 rpm). Thermal treatment of the PNSB biomass preceded by rapid mixing with distilled water resulted in inefficient extraction, which was depicted by the low levels of protein quantified (3–4%). This is probably because the high temperature could not successfully breach the protective outer membrane layer and the thick peptidoglycan layer in the cell wall (Islam et al., 2017). Another study that examined thermal lysis on *E. coli* cells reported optimal membrane permeability and protein release at the same temperature of 90°C with 60 s of heating (Packard et al., 2013). This provides evidence of PNSB’s membrane thermotolerance compared to other gram-negative bacteria. PNSBs have been reported to have high thermotolerance, with some species growing optimally in environments with temperatures as high as 65°C (Charlton, 2002; Okamura et al., 2007). Their comparatively higher heat tolerance is also supported by the presence of heat-tolerant membrane phospholipids (Su et al., 2009; Nagatsuma et al., 2019).

Significantly higher yields (*p* < 0.001) were obtained for all the chemical-assisted thermal treatment groups, with the NaOH-assisted thermolysis proving the most efficient. This is most likely due to increased permeability or softening of cell membranes as a result of chemical pretreatment (Callejo-López et al., 2020). Several other studies have reported that NaOH-aided thermolysis was very effective in breaching the cell walls of other gram-negative bacteria like *E. coli* and microalgae like *Chlorococcum infusionum*, *Scenedesmus* sp. and *Synechococcus* sp., releasing substantial protein (Rausch, 1981; Watson et al., 1987; Ren et al., 2007). However, a downside to combining heating with alkaline treatment is that protein properties could be altered via denaturation or racemization (Schwass and Finley, 1984; Callejo-López et al., 2020). These could significantly affect amino acid characterization, as susceptible amino acids will be underestimated.

**Figure 1:**
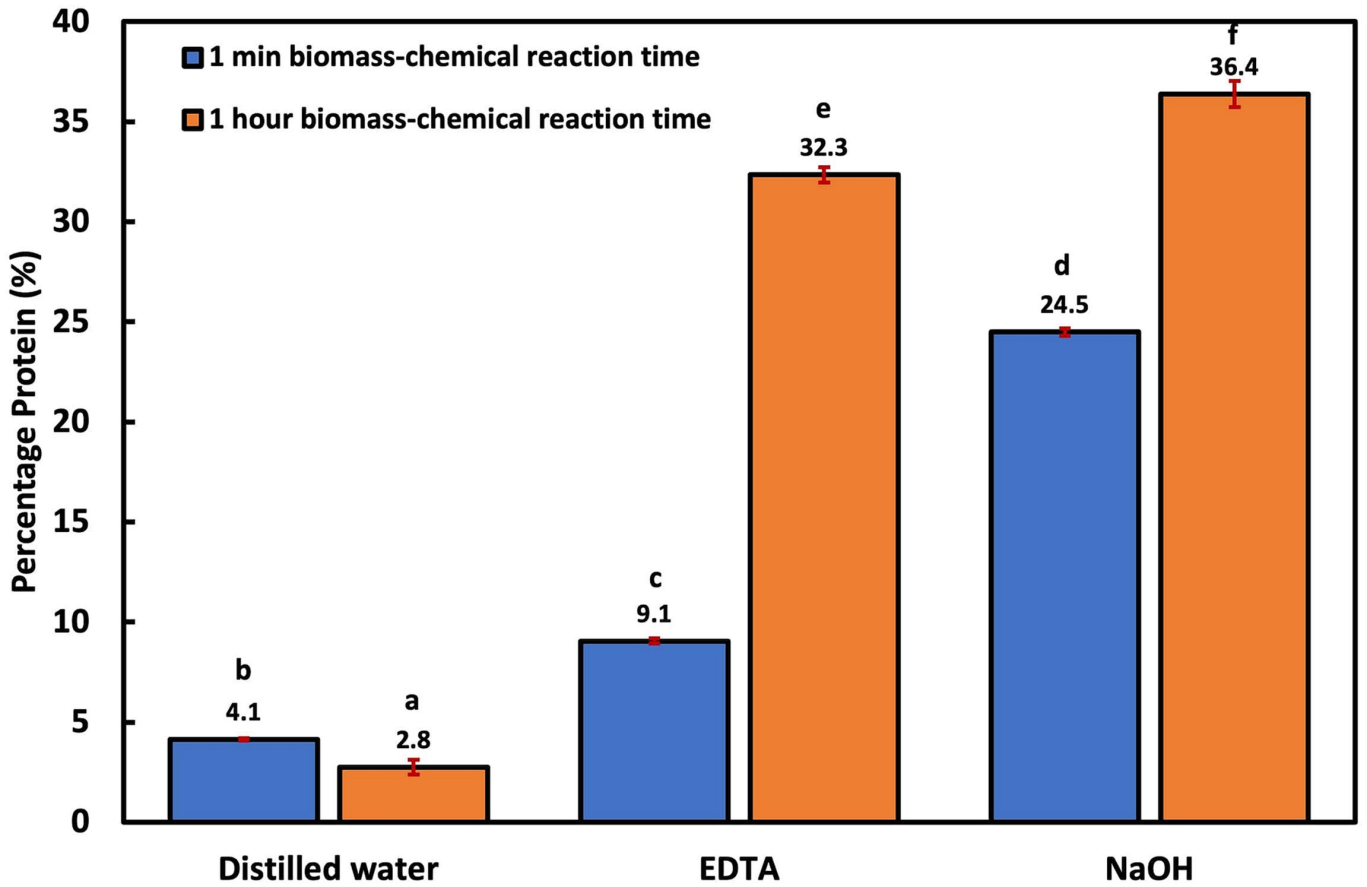
Cellular disruption efficiency using thermal lysis and chemical-aided thermal lysis at no biomass-chemical reaction time and 1 h reaction time.

Overall, the groups with only 1 min biomass-chemical reaction time had significantly less (*p* < 0.006) protein recovery when compared to the groups with 1 h biomass-chemical reaction time (Figure 1). For instance, the EDTA-assisted thermal treatment with 1 min biomass-chemical reaction time had an average protein yield of 9.1 ± 0.1%, while the group with a 1 h reaction time had an average of 32.3 ± 0.4%. A similar pattern was observed with the NaOH-assisted thermal treatment. The benefit of reaction time has also been reported in other studies that employed alkaline-assisted treatments (Gerde et al., 2013; Callejo-López et al., 2019). In one study, protein extraction in *Nannochloropsis* sp. increased significantly as the biomass reaction time with NaOH increased from 1 h to 5 h (Gerde et al., 2013). The reaction time between the chemical and biomass, temperature, and heating duration are important variables to consider for extraction studies because the cell membrane of different cells has varying degrees of permeability. For instance, the highest protein extraction efficiency in recalcitrant microalgae *Scenedesmus* sp. using NaOH-aided thermolysis was reported at 80°C for 90 min, while in more fragile microalgae like *Synechococcus* sp., the best protein yield was reported within 5 to 15 min at 90°C, and in activated sludge, optimal protein extraction is obtained at 100–140°C within 1.5–4 h (Rausch, 1981; Yan et al., 2022). Comparing the chemical-assisted treatment groups, the NaOH-assisted treatment resulted in the highest protein yields both with 1 h (36.4 ± 0.7%) and 1 min (24.5 ± 0.2%) biomass-chemical reaction time, followed by the EDTA-assisted treatment.

### 3.3 Sonication treatment

**Figure 2:**
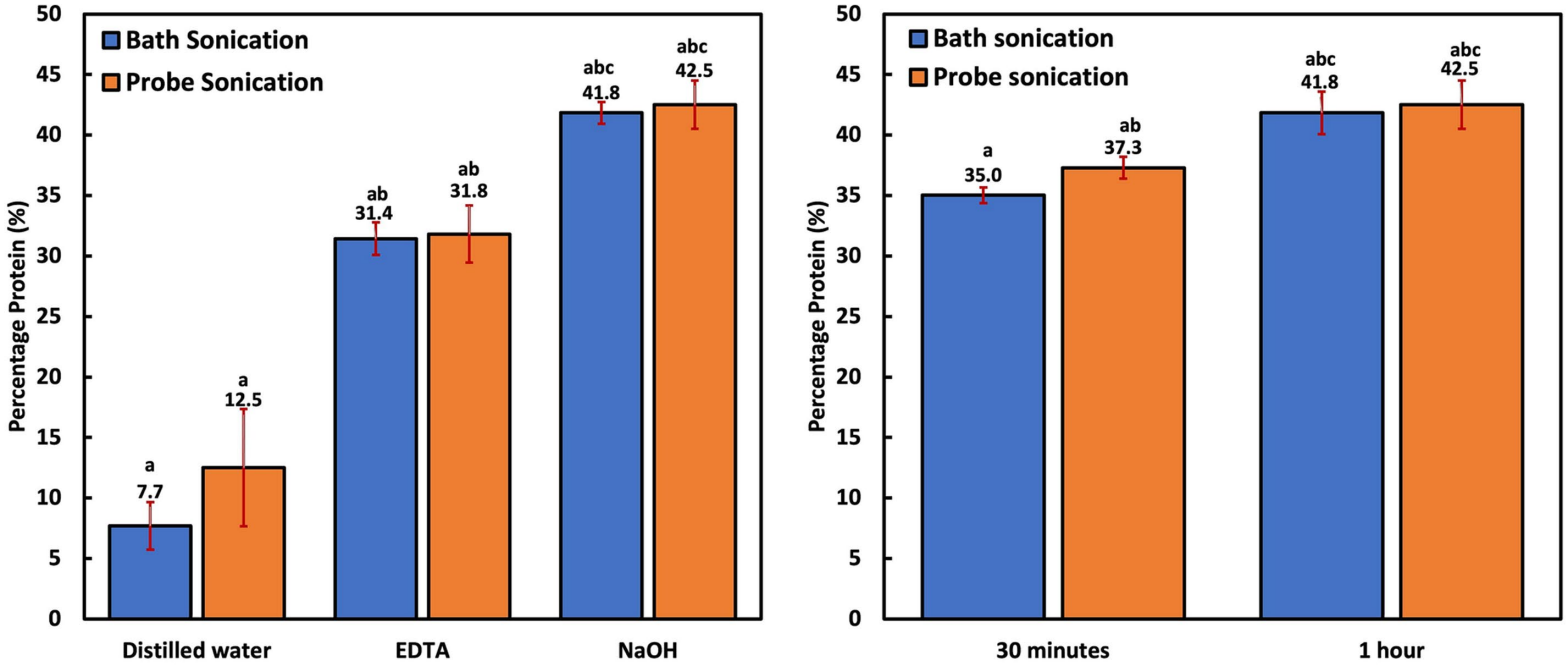
**(A)** Cellular disruption efficiency of bath and probe sonication and chemical-assisted bath and probe sonication; **(B)** Influence of time on NaOH-aided bath and probe sonication.

All experiments in this group were performed after a 1 h reaction time. Protein extraction with bath and probe sonication was more efficient than thermal treatment. However, protein yield (8–13%) was still significantly lower (*p* < 0.001) when compared to the chemical-aided sonication techniques (Figure 2A). PNSB’s resistance to sonication can be attributed to their rigid cell membrane (Weckesser et al., 1995) and small cell size, resulting in a low surface area susceptible to sonic ruptures. The inefficiency of ultrasonication treatment alone for cellular disruption has also been reported in microalgae studies with *Chlorococcum* sp., *Botryococcus* sp., and *C. vulgaris* biomass, also known to have thick cell walls (Lee et al., 2010; Halim et al., 2012). Similar to the thermal treatments, chemical-aided cellular disruption was more effective. Moreover, protein extraction efficiencies were significantly higher (*p* < 0.006) in the NaOH-assisted sonication groups (42–43% biomass protein) compared to the EDTA-assisted groups (31–32% biomass protein). This was also observed in the chemical-aided thermal treatment studies. This could be because proteins are highly solubilized in very alkaline solutions (Gerde et al., 2013), but are more likely because EDTA is an interfering compound (could impede color change) for the modified Lowry assay (Thermo Scientific, 2020). This was confirmed by comparing the impact of distilled water, NaOH and EDTA diluent on preparing 1 mg/mL of albumin standards (BSA). The results revealed that the EDTA diluent group resulted in a significantly lower (*p* < 0.001) BSA concentration of 0.62 ± 0.03 mg/mL, compared to the distilled water (1.07 ± 0.01 mg/mL) and NaOH (1.03 ± 0.02 mg/mL) diluent groups.

Time was revealed to be an important factor for protein recovery. The groups that were subjected to 0.5 h of NaOH-aided sonication had significantly less protein yield (*p* < 0.009) compared to the groups subjected to 1 h of sonication (Figure 2B). This is logical as the PNSB cells are small and difficult to breach, so lengthier treatment time would have better yields. Overall, the probe sonication groups had only slightly higher extraction efficiency (*p* = 1.000) than the bath sonication groups. This is interesting as the bath sonicator transmitted waves at 40 kHz and 80°C instead of the probe’s 20 kHz. However, probe sonication is known to be more targeted, as the sonic waves are in direct contact with the cells. Bath sonication is generally considered less invasive than probe sonication (Mellado et al., 2019). In addition, the high temperature associated with bath sonication could contribute to the slightly lower protein yield. While most proteins can withstand exposure to high temperatures, a few food proteins reportedly degrade at temperatures below 100°C (Huang et al., 2011; Maria, 2019). This could also have impacted the protein yields in the thermal lysis treatment group, as the NaOH-assisted thermal treatment recorded a lower yield (36.4 ± 0.7%) compared to any of the NaOH-assisted sonication groups.

### 3.4 Bead milling

**Figure 3:**
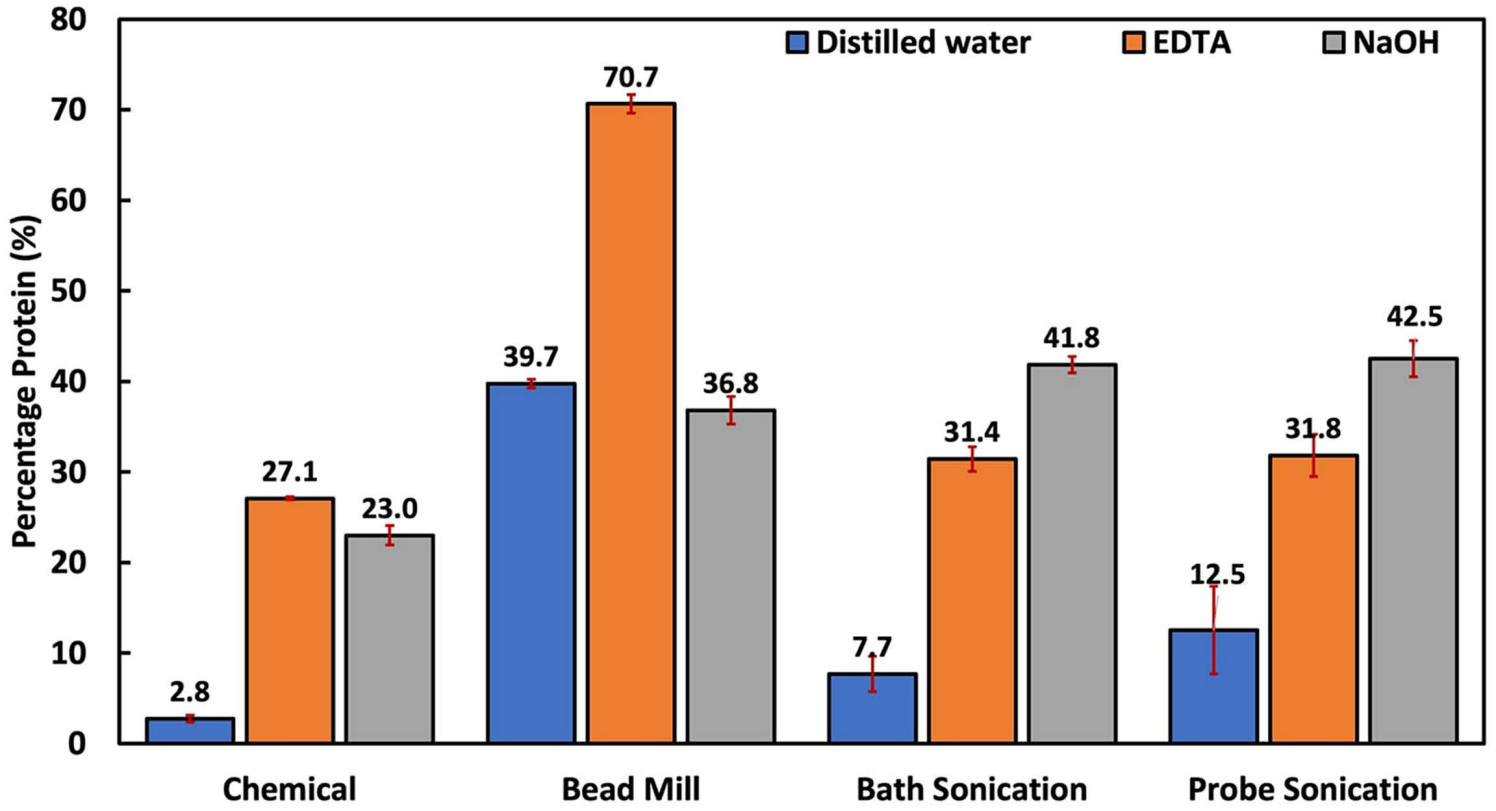
Summary of all treatment techniques at 1 h reaction time. The EDTA-assisted bead mill result is unreliable for reasons summarized in the text and Figure 4.

Bead milling is often regarded as one of the most efficient cellular disruption and extraction techniques (D’Hondt et al., 2017; Hu and Bassi, 2020). As seen in Figure 3, it was the only technique where the protein extraction performance with distilled water was similar to chemical-aided treatment (*p* = 0.268 for NaOH and *p* = 0.137 for EDTA). This is because bead milling depends solely on shear force to breach the cell walls (Islam et al., 2017). This implies that bead milling could be a suitable alternative in scenarios where chemical treatment needs to be avoided. Interestingly, bead milling with DI water had a higher and more stable protein yield compared to the NaOH and EDTA-assisted milling trials, respectively. This was the only technique that yielded such results. This is most probably due to a reduced possibility of protein denaturation and the absence of interfering agents.

**Figure 4:**
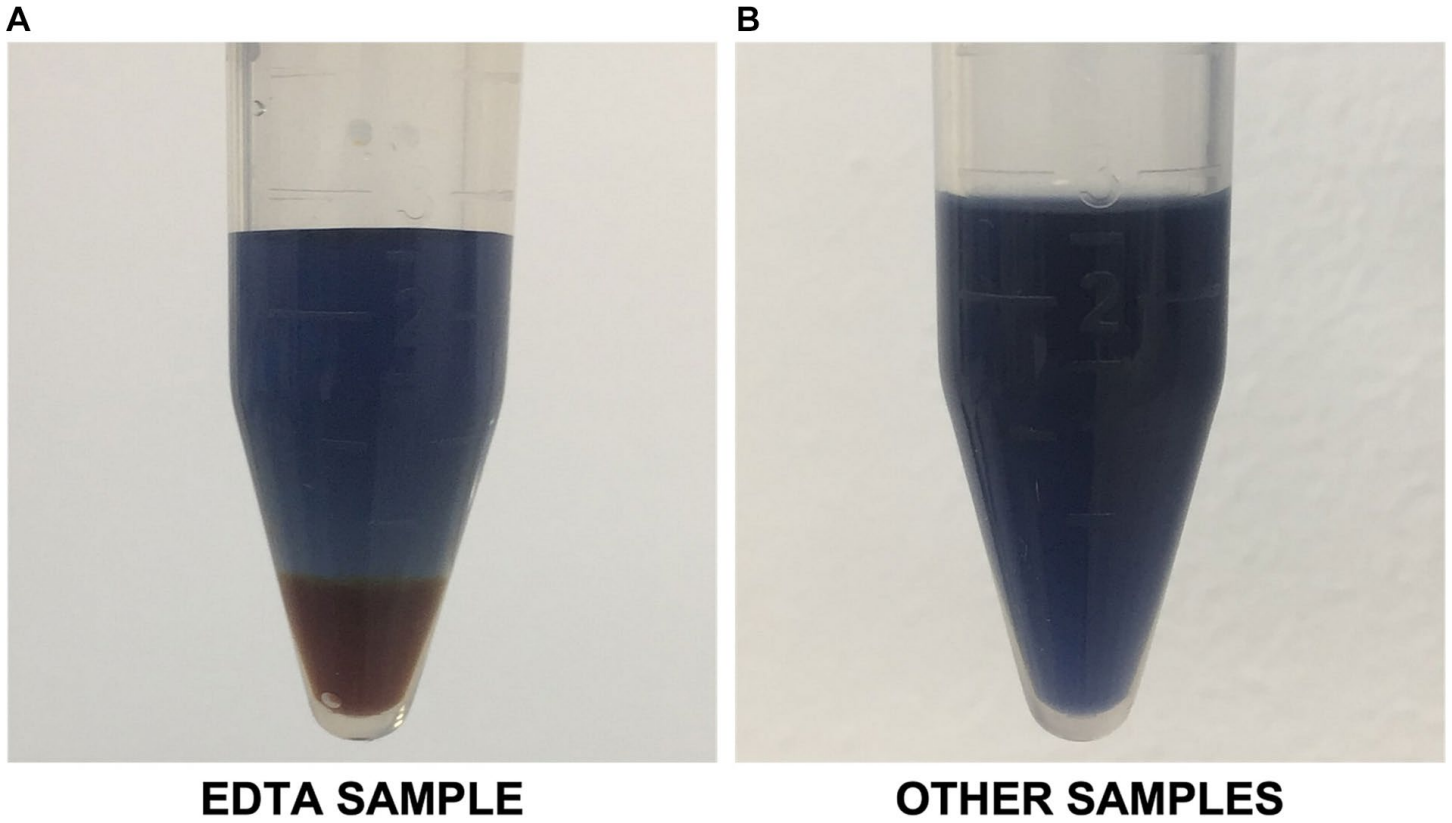
**(A)** EDTA-assisted bead milling sample interfering with modified Lowry reagent; **(B)** Representative image of other samples with no apparent interference.

Hydrophilic bioproducts like proteins are susceptible to degradation due to factors such as high temperature and exposure to solvents, and non-neutral pH solutions (Postma et al., 2016). For example, past studies have reported that alkaline and heat treatments of microbial biomass could alter protein properties via denaturation, racemization, and lysinoalanine formation (Schwass and Finley, 1984; Callejo-López et al., 2019). Moreover, with EDTA primarily functioning as a chelating agent, it could potentially alter the solubility and stability of metalloproteins, leading to their precipitation or denaturation, eventually affecting the overall yield of soluble proteins (Janecki and Reilly, 2005). Results also revealed that the EDTA-assisted bead milling produced a supernatant that interfered with the Lowry reagent. This created an orange-like complex at the bottom of the Eppendorf tubes (Figure 4). The formation of this complex resulted in over-amplified protein quantification results. This is most probably because of EDTA’s chelating properties, possibly reacting with the stainless-steel beads (Oviedo and Rodríguez, 2003).

However, a notable drawback of using a bead mill is the high energy consumption and rise in temperature, which could affect heat-sensitive components like carotenoids and coenzyme Q10 (Hu and Bassi, 2020). Compared to the sonication techniques, the protein extraction efficiency of the bead mill was slightly lower. This is probably because of the 2 mm beads used in this study. Studies have reported that smaller bead sizes (<1 mm) are more efficient for cell lysis (Harrison, 1991; Prapulla and Karanth, 2014). Bead size will likely affect the efficiency of PNSB cellular disruptions because the cells are smaller than most of the microbes studied.

### 3.5 Impact of disruption methods on amino acid characterization

The amino acid characteristics of two of the best treatment methods (bead mill-distilled water and bath sonication-NaOH) were assessed to examine possible differences in the amino acid profiles. As seen in Table 2, all 21 essential amino acids were detected in both treatment groups, confirming the efficacy of the extraction methods. Eight of the amino acids (alanine, arginine, aspartic acid, cysteine, glutamic acid, glutamine, lysine, and threonine) were detected in similar portions, with less than a 1% difference. On the other hand, 11 amino acids (asparagine cystine, histidine, isoleucine, leucine, methionine, phenylalanine, serine, tryptophan, tyrosine, and valine) were detected in higher concentrations (over 10% difference) among the bath sonication treatment group compared to bead mill treatment. In comparison, glycine and proline were detected in higher concentrations in the bead mill treatment group.

**Table 2:** Amino acid characterization of alkali-assisted bath sonication and bead milling with distilled water treatment groups.

Amino acid (g/g)	Bath sonication-NaOH treatment	Bead mill-distilled water treatment	Relative % increase in amino acids for sonication
Alanine	3.31	3.14	5.3
Arginine	1.78	1.84	−3.0
Asparagine	1.41	0.92	**54.1**
Aspartic acid	2.76	2.99	−7.6
Cysteine	0.13	0.14	−4.1
Cystine	0.25	0.20	**23.1**
Glutamic acid	2.28	2.23	2.1
Glutamine	0.61	0.61	−0.3
Glycine	4.39	5.35	**−18.1**
Histidine	0.87	0.79	**11.0**
Isoleucine	1.33	1.15	**16.0**
Leucine	3.40	2.69	**26.6**
Lysine	1.98	2.08	−4.8
Methionine	0.63	0.55	**15.8**
Phenylalanine	1.68	1.35	**24.2**
Proline	2.48	2.88	**−14.0**
Serine	4.30	3.81	**13.0**
Threonine	3.11	2.93	6.2
Tryptophan	0.65	0.49	**31.5**
Tyrosine	1.41	1.18	**18.9**
Valine	3.03	2.38	**26.9**

Bolded values are where extracted amino acid varied by 10% or more from the alternative extraction method.

Overall, the results indicate that extraction efficiency was higher in the alkali-assisted bath sonication group. This is likely due to the solubility characteristics and chemical environments facilitated by NaOH in the sonication process. The alkali solution may enhance the solubility and extraction of certain amino acids, increasing their concentrations in the resulting solution. For example, past literature has reported that amino acids like alanine, leucine, isoleucine, valine, phenylalanine, tyrosine, and serine are more soluble in alkaline solutions compared to neutral pH (Dalton et al., 1930; Needham et al., 1971; Pradhan and Vera, 1998; Tseng et al., 2009). This is because amino acids are least soluble close to their isoelectric point (no net electric charge), which is facilitated by the distilled water treatment (Shaw et al., 2001; Tseng et al., 2009). All the amino acids expressed in higher concentrations in the alkali-assisted group have their isoelectric points between 5.41 and 6.01 (Liu et al., 2004). Thus, alkali-assisted extraction methods will favor the detection of such amino acids.

Furthermore, the sonication process may preserve the stability of sensitive amino acids like tryptophan, threonine, cystine, and serine, which are prone to degradation or modification during bead milling (ThermoScientific, 2001). In addition, methionine, an amino acid susceptible to oxidation, could be better preserved and extracted using sonication, since bead milling can induce oxidation reactions via mechanochemistry (ThermoScientific, 2001; Métro et al., 2014). The comparatively higher solubility of glycine and proline in water probably contributed to their improved extraction efficiency under distilled-water bead milling than alkali-assisted bath sonication (Ji and Feng, 2008; Othman et al., 2018). Besides solubility characteristics, protein degradation could potentially contribute to the differences between both groups (Goldberg, 2015). Bead milling is a highly disruptive technique, potentially resulting in protein degradation because of high energy dissipation and fluctuating temperatures during milling (van Gaver and Huyghebaert, 1991; Phillips et al., 2021). On the other hand, sonication is generally less disruptive at reasonable intensities, leading to lower chances of protein denaturation. This hypothesis was supported by results in a recent study that examined total protein extracted from the microalgae *Arthrospira plantensis*. In that study sonication had the highest yield, which was more than double the protein yield obtained from beadmilling (Spínola et al., 2023).

### 3.6 Efficacy of extraction methods for CoQ10

**Figure 5:**
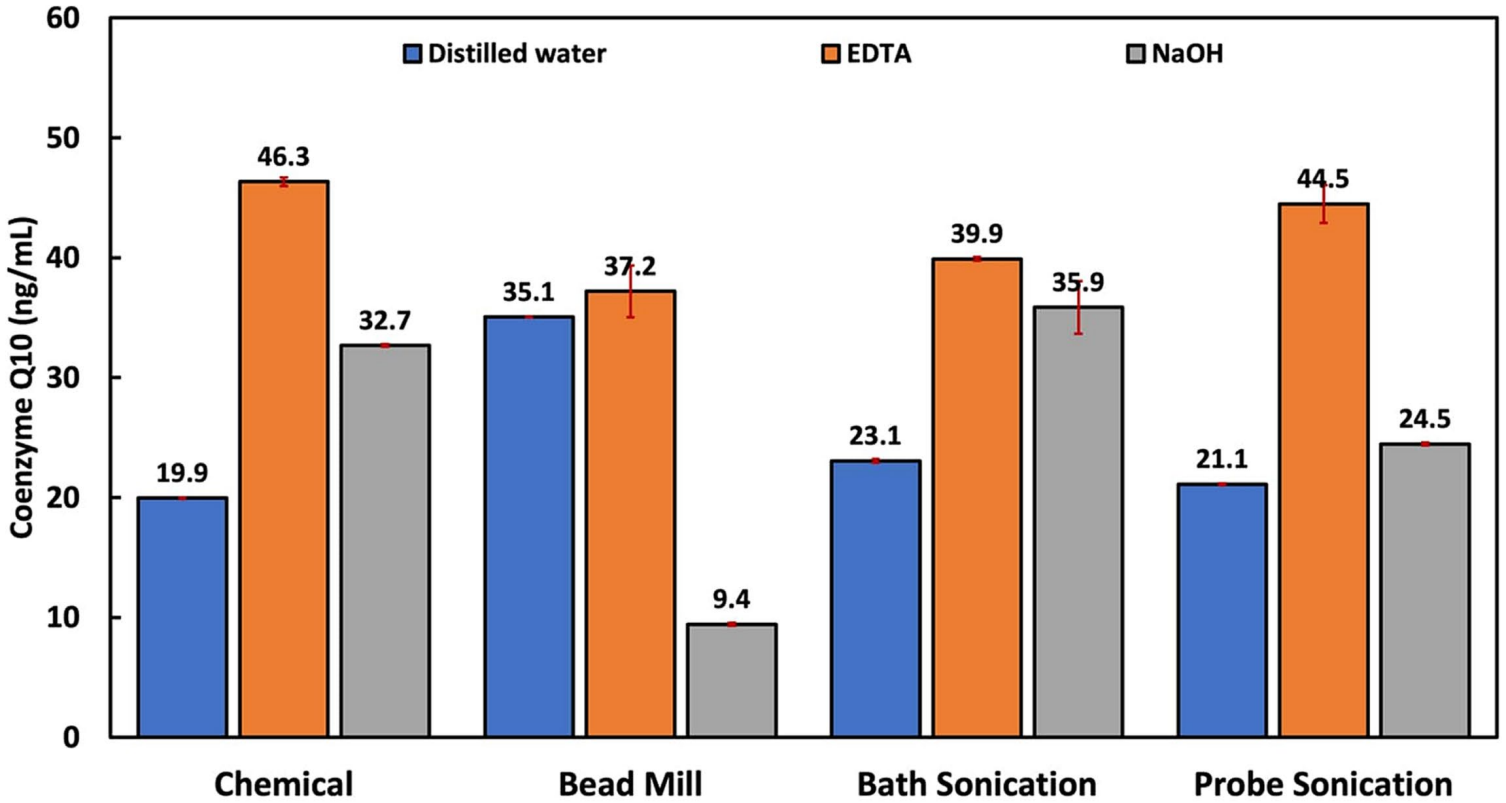
Influence of cellular disruption techniques on coenzyme Q10 extraction and quantification.

CoQ10 is fat-soluble and is localized in the cell membrane around the lipid bilayer, where it functions as a cofactor for the electron transport chain (Aussel et al., 2014). Using minimum force during cell lysis is recommended to derive high CoQ10 yields due to its sensitivity (Wu and Tsai, 2013). Thus, enzyme and solvent extraction methods have been recommended over more disruptive and non-specific lysis methods like mechanical and physical disruption. The highest CoQ10 content was obtained via chemical treatment with EDTA, followed by EDTA-assisted probe sonication, EDTA-assisted bath sonication and EDTA-assisted bead milling (Figure 5). EDTA treatment methods clearly outperformed distilled water and NaOH treatment techniques. This is probably due to the EDTA’s function as an emulsifier and solubilizer, considering CoQ10’s hydrophobicity and the strong hydrophilic nature of distilled water and NaOH.

A study comparing several extraction methods reported that the best single-step extraction was achieved using ethanol capable of both cell disruption and CoQ10 extraction (Wu and Tsai, 2013). Ethanol has been reported to be an ideal solvent for extraction due to the high solubility/stability of CoQ10 (Bule and Singhal, 2012). In this study, EDTA had similar features as it induced cell lysis and solubilized CoQ10, as opposed to NaOH, which induced cell lysis but could not efficiently extract the CoQ10. Another factor possibly responsible for EDTA’s superior performance is the lower pH of the EDTA solution (pH 8) compared to the NaOH solution (pH 13). This is due to the structural alterations sustained in alkaline media, leading to the formation of other quinone compounds (Bogeski et al., 2011; Petrova et al., 2014). In a previous study, the NaOH pretreatment method was also associated with poor CoQ10 yields, even though it was followed by a solvent extraction process (Wu and Tsai, 2013). This supports the notion that CoQ10 could be destabilized under basic conditions.

The significantly higher CoQ10 extraction performance in the EDTA treatment and EDTA-aided probe sonication compared to the other EDTA-based treatment methods could be due to the effect of temperature on CoQ10. Past extraction studies have reported that CoQ10 is light, temperature and pH sensitive, and is stable at a temperature range of 4 to 60°C and a pH range of 6 to 9 (Fir et al., 2009; Nguyen et al., 2015). In one study, the highest CoQ10 extraction was obtained at 40°C and continued to reduce as temperature increased (Bule and Singhal, 2012).

### 3.7 Overview of extraction techniques

Overall, the hybrid cellular extraction methods involving chemical–physical/mechanical pretreatments had the best protein extraction efficiency. Considering only chemical pretreatment, EDTA (27%) was associated with significantly higher protein yield than NaOH (21%) after a 2 h reaction time at 100 rpm. However, a longer reaction time and higher mixing velocity could increase protein yield. Considering mechanical and physical methods, only bead milling achieved an optimal protein yield (40%), which was almost at par with NaOH-bath sonication (42%) and NaOH-probe sonication (43%). When used solely, protein extraction efficiencies of thermal, bath sonication and probe sonication were poor: 3, 8, and 13%, respectively. This was attributed to PNSB’s small cell size and rigid cell wall. Thus, for PNSB studies, it is advised to employ highly disruptive treatment techniques like bead beating or chemical-assisted physical/mechanical methods to achieve optimal protein recovery.

On the other hand, CoQ10 extraction was dependent on the disruption/extraction solution. The highest CoQ10 yields were obtained when EDTA was employed due to EDTA’s superior ability to solubilize CoQ10 over distilled water and NaOH. Other suspected contributing factors were temperature and pH, which are known to destabilize CoQ10 at elevated levels. Thus, pure chemical treatment with EDTA and EDTA-assisted probe sonication is recommended, as the two groups had the highest extraction efficiencies.

When the extraction efficiencies of both protein and CoQ10 are considered, the utilization of the EDTA pretreatment method and EDTA-assisted probe sonication pretreatment method can be considered. Finally, regarding energy considerations, the single EDTA chemical treatment method is preferred as it requires negligible energy for mixing. Among the physical/mechanical techniques, NaOH-assisted probe sonication generated the least energy (0.15 kWh) as it transmitted waves at a frequency of 20 kHz, compared to the bath sonication (0.665 kWh) that transmitted a frequency of 40 kHz and heating at 80°C. As expected, the highest energy consumption was associated with the high-energy bead mill (3.1 kWh).

## 4 Conclusion

The efficient disruption of PNSB for protein/coQ10 quantification presents a unique set of challenges when compared to more well-studied organisms like gram-negative bacteria such as *E. coli* or autophototrophic Cyanobacteria. The distinct cellular characteristics of PNSB, notably their small size, have necessitated exploring disruption methods tailored to their specific properties. Here are the key findings from our study.

Unlike other commonly explored microbes, traditional techniques like chemical, thermal, and alkaline-assisted thermal treatment have proven ineffective for PNSB.

Likewise, probe and bath sonication, known to be efficient disruption methods for other biomasses (Liu et al., 2013; Islam et al., 2017), do not yield satisfactory results for PNSB.

The NaOH-aided sonication treatment was the most effective protein extraction technique for PNSB biomass.

Excluding bead milling, the chemical-assisted mechanical/physical techniques achieved significantly better protein recovery than singular treatment techniques.

The reaction time between the chemical and biomass significantly affected cell disruption efficiency. For example, alkali-assisted sonication required over 30 min of reaction time, significantly longer than the reaction time employed for common bacteria and microalgae disruption techniques (5 to 25 min) (Monique et al., 2008; Sierra et al., 2017).

NaOH was preferred to EDTA for protein quantification as EDTA was identified to interfere with the modified Lowry protein assay, resulting in significantly lower protein concentrations.

Amino acid characterization of two of the best methods (NaOH-aided sonication and bead milling) revealed that about 50% of the amino acids were detected in higher concentrations in the NaOH-aided sonication. This was likely linked to the increased solubility of amino acids in alkaline conditions and the non-disruptive mechanism of cell lysis employed by sonication.

For CoQ10 extraction, chemical treatment with EDTA was preferable, while harsher physical and mechanical treatments were harmful. The efficiency of coenzyme Q10 extraction was also suspected to be adversely influenced by high temperature and pH.

## Data availability statement

The raw data supporting the conclusions of this article will be made available by the authors, without undue reservation.

## Author contributions

OW: Conceptualization, Data curation, Formal analysis, Investigation, Methodology, Writing – original draft, Writing – review & editing. NR: Investigation, Methodology, Writing – review & editing. PW: Formal analysis, Investigation, Writing – review & editing. PT: Formal analysis, Investigation, Resources, Writing – review & editing. GM: Methodology, Project administration, Resources, Supervision, Writing – review & editing. HM: Conceptualization, Funding acquisition, Methodology, Project administration, Resources, Supervision, Writing – review & editing.
